# Mental Health among Former Child Soldiers and Never-Abducted Children in Northern Uganda

**DOI:** 10.1100/2012/367545

**Published:** 2012-04-30

**Authors:** Ughetta Moscardino, Sara Scrimin, Francesca Cadei, Gianmarco Altoè

**Affiliations:** Department of Developmental and Social Psychology, University of Padova, via Venezia 8, 35131 Padova, Italy

## Abstract

The present study aimed to evaluate posttraumatic stress symptoms, psychological distress, and emotional and behavioral problems in former Ugandan child soldiers in comparison with civilian children living in the same conflict setting. Participants included 133 former child soldiers and 101 never-abducted children in northern Uganda, who were interviewed about exposure to traumatic war-related experiences, posttraumatic stress symptoms, psychological distress, and emotional and behavioral problems. Results indicated that former child soldiers had experienced significantly more war-related traumatic events than nonabducted children, with 39.3% of girls having been forced to engage in sexual contact. Total scores on measures of PTSD symptoms, psychological distress, and emotional and behavioral problems were significantly higher among child soldiers compared to their never-abducted peers. Girls reported significantly more emotional and behavioral difficulties than boys. In never-abducted children, more mental health problems were associated with experiencing physical harm, witnessing the killings of other people, and being forced to engage in sexual contact.

## 1. Introduction

Everyday worldwide, hundreds of thousands of children are forced to serve as soldiers in armed conflicts becoming the recipients and perpetrators of violence and thus experiencing a great number of atrocities [1]. Since the late 1980s, Northern Uganda has been plagued by the armed conflict between government forces and the opposition Lord's Resistance Army (LRA), one of the biggest world recruiters of child soldiers. About 25,000 boys and girls were abducted by the LRA from the beginning of the conflict [2]. During attacks against Uganda's Acholi people, children in the LRA were forced to participate in combat and to carry out raids, kill and mutilate other child soldiers and civilians, and loot and burn houses. Children had to kill relatives, were beaten or mutilated either as punishment or if physically unable to keep up with their unit, and girls were routinely raped, given as wives to commanders, and bore children in captivity [3, 4].

Undoubtedly, being a former child soldier is related to a number of traumatic experiences which have detrimental effects on children's mental health [5]. Previous research conducted in the North Ugandan context revealed high rates of posttraumatic reactions among children who had been abducted by the LRA [6, 7]. Yet, there is a paucity of studies comparing the mental health status of former child soldiers with that of children living through war without being recruited into armed groups. Kohrt and colleagues [8] compared differently exposed groups in Nepal and reported greater severity of mental health problems in former child soldiers compared with children never conscripted by armed groups, also after controlling for trauma exposure. However, it is not yet clear what aspects of the child soldier experience contribute to the poorer mental health outcomes in this population. Specifically, the extent to which this difference may be related to the impact of other experiences, such as sexual violence, on children's psychosocial well-being is still unknown. As Betancourt [9] noted, children's exposure to war-related trauma varies greatly as a function of context and other individual factors such as gender. Given that effective intervention strategies need to be culturally sensitive and respectful of the social ecology, it is important to report on the mental health of former Ugandan child soldiers in comparison with children never abducted by the LRA to address the specific problems faced by this war-affected population.

Therefore, the first objective of this study was to compare the mental health status of former child soldiers with that of children growing up in the same active conflict setting, but who had not been abducted by armed groups in northern Uganda. We also aimed to assess whether the impact of soldiering differed between girls and boys. The second objective was to investigate the association between single war-related traumatic events and psychiatric problems among former child soldiers and never-abducted children in an attempt to identify the most traumatizing experiences that contribute to increased psychopathology.

## 2. Patients and Methods

### 2.1. Setting and Participants

This research is part of a larger, mixed-method study conducted between August 2006 and June 2007 on the mental health effects of the armed conflict in northern Uganda. The aim of this study was to assess posttraumatic stress symptoms, psychological distress, and emotional and behavioral problems among former Ugandan child soldiers in comparison with children never abducted by the northern Ugandan Lord's Resistance Army (LRA).

From the beginning of the conflict, Joseph Kony's rebellion movement, the LRA, committed numerous human-rights abuses and atrocities against the civilian population of northern Uganda. Tens of thousands of people have been killed and mutilated, hundreds of thousands displaced, and farming activities and livestock have been totally disrupted. The abduction of children is the main method of recruitment of the LRA and about 90% of recruits are children. Armed conflict between government forces and the opposition LRA in northern Uganda continued until early 2006, yet the government's strategy of pursuing a military solution to the conflict contributed to humanitarian suffering and abuses against civilians. More than 1.4 million people live in camps for internally displaced persons (IDPs) which are characterized by acute overcrowding and lack of accommodation, medical care, and nutrition [10].

Data were collected based on a 2-stage process. In the first stage, ethnographic methods were used to obtain qualitative information on the Acholi culture and on the consequences of the armed conflict for the civilian population. Participant observation, interviews, and focus groups with key informants (e.g., teachers, caregivers, and aid workers) as well as youths living in camps for internally displaced persons near Gulu town were conducted in collaboration with community workers and local leaders. In the second stage, we randomly selected adolescents from four secondary schools located in the Gulu district. This area has been at the epicenter of the northern Ugandan war [11]. Youths were included if they were 18 years or younger and had been living in the area for the last year. Study participants were identified by selecting every second child listed on the registration books provided by the school principals of the four schools. The cross-sectional survey data was collected by a trained clinical psychologist (F. Cadei), who spent a prolonged period of time (>1 year) working with Ugandan youth in the context of a humanitarian project and thus was knowledgeable about the local culture and language. The data were collected between February and May 2007 under supervision of the first and the second author (U. Moscardino and S. Scrimin). The research was conducted in accordance with the principles embodied in the Declaration of Helsinki, and local ethical and legal requirements were followed. The principals of the schools that participated gave their written approval for the study. After obtaining voluntary verbal consent from participants and their guardians, adolescents were interviewed individually in private locations to assure confidentiality. Subjects' interviews lasted approximately 60–90 minutes, and items were read to children to ensure comprehension; when necessary, an interpreter was available during the interview. A total number of 238 youths aged 14–18 years were invited to take part in the study; of these, 234 agreed to participate.

### 2.2. Measures

Adolescents were interviewed using standardized questionnaires to evaluate posttraumatic stress symptoms, psychological distress, and emotional and behavioral problems. Instruments were selected for their well-established psychometric properties as well as their easiness of comprehension to reduce participant burden. Prior to data collection, all questionnaires were translated from English to Acholi and back translated by local professional translators to ensure cultural sensitivity and appropriateness. In addition, we conducted several focus groups with bilingual mental health professionals who were asked to judge the accuracy of measures [12]. Teachers, local experts, and adolescents not enrolled in the study also reviewed the questionnaires for their cultural sensitivity, appropriateness, and noninvasiveness.

Basic demographic information was obtained on adolescents' age, sex, family composition, educational level, age at the time of abduction, length of abduction, and current living arrangement. Based on qualitative interviews with local experts as well as previous work conducted with former child soldiers in northern Uganda [6, 7], a list of 10 yes/no questions was developed to assess exposure to war-related traumatic experiences in youths.

Posttraumatic stress symptoms were assessed via the Impact of Events Scale Revised (IES-R) [13]. The IES-R is a 22-item self-report scale, parallel to the DSM-IV criteria for PTSD, that includes three subscales: avoidance (8 items), intrusion (7 items), and hyperarousal (7 items). Respondents are asked to rate the frequency of PTSD symptoms in the past month from 0 to 4. A total score is obtained by summing all of the scores, with possible scores ranging from 0 to 88. In this study, we determined a total severity score and used a cutoff score of greater than 24 to identify adolescents with clinically significant symptoms [6]. The IES-R has been widely used in diverse cultural settings [14], demonstrating good reliability and validity also for Ugandan youth [6]. The internal consistency of the scale in this study was Cronbach *α* = 0.87.

Adolescents' psychological distress was assessed via the Brief Symptom Inventory 18 (BSI-18) [15], an 18-item version of the 53-item Brief Symptom Inventory [16]. The questionnaire includes three subscales assessing symptoms of depression, anxiety, and somatization.

Adolescents indicated how frequently they had been distressed or bothered by symptoms in the past months using a scale ranging from 0 to 4. A global severity index (GSI) is obtained by summing all of the scores, with possible scores ranging from 0 to 72. In this study, we computed a total severity score and used a T score of 63 or higher to classify a child as having clinically significant psychological distress symptoms [15]. The BSI-18 has been widely used with psychiatric, medical, and community populations, demonstrating good internal consistency and reliability also for adolescent samples [17]. Reliability of the scale in this study was high (Cronbach *α* = 0.88).

The children's emotional and behavioral difficulties were assessed using the Strength and Difficulties Questionnaire (SDQ), a 25-item self-report measure that can be administered to parents and teachers of 4- to 16-year olds and to 11- to 16-year olds themselves [18]. The questionnaire includes 5 subscales of five items each, generating scores for emotional symptoms, conduct problems, hyperactivity inattention, peer problems, and prosocial behavior [19]. Items are rated on a scale ranging from 0 (not true) to 2 (certainly true). A total difficulty score is obtained by summing all of the scale scores but the last, with possible scores ranging from 0 to 40. In the present study, we used the total difficulty score and a cutoff score of 16 to identify youths with clinically significant mental health problems [8]. The SDQ has been used previously in a variety of cultures, including northern Uganda [20]. In this study, internal consistency measured by Cronbach's *α* for this score was 0.67.

### 2.3. Data Analysis

Descriptive statistics were used to present the characteristics of the sample. To analyze differences between the groups on sociodemographic variables, we used the *χ* ^2^ test for categorical variables and the *t*-test for continuous variables. We did univariate logistic regression to model the association between child soldier status, sex, and exposure to war-related traumatic events. Group differences on likely rates of psychiatric disorders were examined via odds ratios (ORs). We used multivariate analyses of covariance to investigate the association between independent (child soldier status, sex, and age) and dependent variables (PTSD symptoms, psychological distress, and emotional and behavioral difficulties). In these analyses, we used the total psychopathology scores rather than cutoff scores because Kolmogorov Smirnov test showed that all outcome variables were normally distributed (all *Ps* > .05), and thus dichotomizing these variables would have decreased the explanatory power of the analyses. Wilk's lambda (Λ) was the multivariable test of significance. To investigate the associations between trauma exposures and the total psychopathology scores separately for each group, we used Pearson product-moment correlations.

## 3. Results

### 3.1. Sociodemographics and Exposure to Traumatic Events

234 Acholi adolescents participated in the study: 133 (57%) who had been abducted by the LRA, and 101 (43%) controls who had not. Former child soldiers reported having been abducted by armed forces at a young age (mean [SD], 11.9 [2.9]; range, 5–17 years) and having served a mean of 24 months (SD, 22; range, 0–118 months). None of the former child soldiers were actively participating with an armed group at the time of the study.

As shown in Table 1, the sample was composed of approximately equal numbers of boys (52.1%) and girls (47.9%). The mean age of the respondents was 16.7 years (SD, 1.2 years). The two groups did not differ in sex and mean age. Seventy-six percent of youths lived in camps, 19% in their homes, and 4% reported other living arrangements (e.g., relatives' homes). More former child soldiers lived in camps (*P* = .004) compared to never-abducted children. Overall, 105 (44.9%) adolescents were orphans (46.3%). However, former child soldiers more frequently reported the loss of the mother (*P* < .001) or father (*P* < .001) than their nonabducted peers.

On average, adolescents had been exposed to 5 different war-related traumatic events (SD, 3.3; range, 0–10). In logistic regression analyses, all exposures were significantly predicted by former child soldier status (see Table 1), providing evidence that the objective features of exposure were intrinsically related to the grouping variable of being or not being a former child soldier. The most frequently reported traumatic experiences in former child soldiers were having to carry heavy loads (96.2%), having to loot properties (80.9%), and being injured (79.7%). More than half of these youths were forced to participate in fights (75.8%) and saw someone being killed in their abduction (69.2%). Sixty-one participants (51.1%) had to drink their own urine, and 60 (45.1%) were forced to engage in sexual contact. Hence, the level of multiple exposure to traumatogenic elements was very high among former child soldiers compared to children never abducted by armed groups. In addition, boys were more likely than girls to having been beaten (OR, 0.47; 95% CI, 0.26–0.86) and having been forced to fight (OR, 0.53; 95% CI, 0.27–0.99), whereas girls were more likely to having been forced into sexual contact compared to boys (OR, 1.80; 95% CI, 1.01–3.18).

### 3.2. Mental Health among Former Child Soldiers and Never-Abducted Children

According to previously established cutoff scores, prevalence rates of mental health problems were high in both groups. Overall, 217 (92.7%) adolescents met PTSD symptom criteria on the Impact of Event Scale Revised, 205 (87.6%) were in the likely clinical range for psychological distress on the Brief Symptom Inventory 18, and 124 (53.0%) reported significant emotional and behavioral difficulties on the Strength and Difficulties Questionnaire. More former child soldiers scored above the cutoff scores for all mental health outcomes in comparison with non-abducted children, including PTSD symptoms (*n* = 130 child soldiers [97.7%] versus *n* = 87 never-abducted children [86.1%]) (OR, 6.97; 95% confidence interval [CI], 1.95–24.98), psychological distress (*n* = 124 [93.2%] versus *n* = 81 [80.2%]) (OR, 3.40; 95% CI, 1.48–7.84), and behavioral and emotional problems (*n* = 90 [67.7%] versus *n* = 34 [33.7%]) (OR, 4.12; 95% CI, 2.38–7.15).

Table 2 shows the total scores on the mental health measures across the two groups. In multivariate analysis of covariance, we recorded significant differences in total symptom scores between former child soldiers and their non-abducted counterparts (Wilk's Λ = 0.83, F_3,227_ = 15.58, *P* < .001) as well as between boys and girls (Wilk's Λ = 0.97, F_3,227_ = 2.72, *P* < .05). Follow-up univariate analyses indicated that former child soldiers scored significantly higher on PTSD symptoms, psychological distress, and emotional and behavioral problems compared to never-abducted children. With regard to gender differences, girls reported significantly more psychological difficulties than boys. We found no significant group by sex interaction (Wilk's Λ = 0.99; F_3,227_ = 0.49; *P* > .05), and age was not associated with any of the outcome variables (Wilk's Λ = 1.00; F_3,227_ = 0.27; *P* > .05).

Correlations between single traumatic exposures and mental health problems are reported in Table 3. Overall, we recorded more significant correlations in never-abducted children compared to former child soldiers. In the latter group, drinking urine was positively correlated with BSI-18 score (*P* = .001) and SDQ score (*P* = .001). The death of mother or father, age at abduction, and period served in the LRA were not associated with any of the outcome variables. In non-abducted children, more PTSD symptoms were positively correlated with having to carry heavy loads (*P* = .006), being seriously beaten (*P* = .009), getting injured (*P* = .001), and witnessing someone being killed (*P* = .005). Similarly, total psychological distress was associated with being seriously beaten (*P* = .001), getting injured (*P* < .001), witnessing someone being killed (*P* < .001), killing someone personally (*P* = .002), having to drink urine (*P* = .009), having to punish other children (*P* = .002), and being forced to engage in sexual contact (*P* < .001). No significant association was found between traumatic exposures and total SDQ score.

## 4. Discussion

This study presents the results from a cross-sectional field study assessing posttraumatic stress reactions, psychological distress, and emotional and behavioral problems among former Ugandan child soldiers and children who had never been abducted by the LRA. Although previous research showed extremely high rates of posttraumatic stress reactions in this population, few studies have investigated former child soldiers' mental health problems in comparison with never-abducted children living in the same conflict setting.

At the time of this research, more than two-thirds (84.6%) of former child soldiers were living in camps for internally displaced persons, and 53.4% reported the death of both parents, thus suggesting that these youths were experiencing very difficult life circumstances despite not being involved in any armed group at the time of data collection. Former child soldiers also had experienced significantly more war-related traumatic events compared to non-abducted children, providing evidence that the objective features of exposure were intrinsically related to former soldier status. In addition, 39.3% of girls reported having been forced to engage in sexual contact, and both sexes had been equally exposed to combat-related traumatic events such as killing someone, witnessing someone being killed, and having to punish other children. These data support previous reports indicating that girl soldiers in the LRA were used for military purposes as well as for sexual slavery [2].

Overall, prevalence rates of mental health problems were high in both groups (>80% for PTSD and psychological distress, >30% for conduct problems). This pattern may be explained by the highly disruptive effect of political violence at multiple levels of the child's environment (i.e., family, peers, and community), since many youths experienced the loss of friends and family members despite not being directly involved in the LRA. Indeed, previous studies suggest that all children living in war zones are in need of special psychosocial intervention [8, 9, 20]. However, more former child soldiers than never-abducted children were in the likely clinical range for PTSD symptoms, psychological distress, and conduct problems according to previously established cut-off scores. The difference in mental health outcomes between child soldiers and never-conscripted children can be explained by greater exposure to traumatic events among child soldiers. Yet, the lack of association between single traumatic events and mental health problems in this group revealed that the number of traumatic experiences and the type of trauma experienced seemed to have little effects on former child soldiers' psychopathology. This finding is consistent with previous research suggesting that the circumstances in which these youths had to survive are traumatizing in themselves [6, 8]. Patterns of correlations among non-abducted children showed that experiencing physical harm, witnessing the killings of other people, and being forced to engage in sexual contact were related to more severe PTSD symptoms and increased psychological distress.

The study has some limitations, including the reliance on self-report measures originally developed in Western countries, the absence of detailed measurements of other potentially relevant variables (e.g., drug and/or alcohol use, economic status, and reintegration difficulties), and lack of information on prewar status of the children. Nonetheless, our results have several implications for clinicians and health professionals working with war-affected youth. First, the high prevalence rates of mental health problems in this population support the idea that interventions should be directed to all children, regardless of their soldier status. Second, girls are in need of specific programs aimed at reducing psychological distress related to forced involvement in sexual activities. Third, adolescents may benefit from school-based psychoeducational interventions focusing on the promotion of effective coping strategies to reduce posttraumatic symptoms. However, a primary concern is to prevent violence and human right abuses as well as facilitate youths' reintegration in their communities, since former child soldiers often face high levels of discrimination and social stigma due to the atrocities committed during their association with armed forces. 

## Figures and Tables

**Table 1:** Sociodemographic characteristics and war-related traumatic experiences of former child soldiers and children never abducted by the Lord's Resistance Army^a^.

Characteristics	Total	Former Child	Never Abducted	*P*-Value^b^
		Soldiers	Children	
	(*n* = 234)	(*n* = 133)	(*n* = 101)	
Sex				.48
Boys	122 (52.1)	72 (54.1)	50 (49.5)	
Girls	112 (47.9)	61 (45.9)	51 (50.5)	
Age, mean (SD) [range], y	16.7 (1.2) [14–18]	16.8 (1.2) [14–18]	16.6 (1.3) [14–18]	.31
Living arrangement				.004
Camp for IDPs	162 (69.2)	104 (84.6)	58 (65.2)	
Home	41 (17.5)	16 (13.0)	25 (28.1)	
Other	9 (3.8)	3 (2.4)	6 (6.7)	
Father alive				<.001
Yes	172 (73.5)	20 (15.3)	36 (37.1)	
No	56 (23.9)	111 (84.7)	61 (62.9)	
Mother alive				<.001
Yes	119 (50.9)	51 (38.9)	60 (60.6)	
No	111 (47.4)	80 (61.1)	39 (39.4)	
				**OR (95%** **CI)^c^**
Traumatic experience				
Having to carry heavy loads	170 (72.6)	127 (96.2)	43 (42.6)	35.04 (13.10–93.71)
Being seriously beaten	133 (56.8)	102 (77.9)	31 (30.7)	8.24 (4.50–15.08)
Getting injured	158 (67.5)	106 (79.7)	52 (51.5)	3.67 (2.06–6.53)
Witnessing someone being killed	121 (51.7)	90 (69.2)	31 (30.7)	5.08 (2.89–8.92)
Killing someone personally	68 (29.1)	61 (46.6)	7 (6.9)	11.67 (5.03–27.06)
Having to drink urine	77 (32.9)	67 (51.1)	10 (9.9)	9.67 (4.61–20.27)
Having to loot properties	129 (55.1)	106 (80.9)	23 (23.0)	14.16 (7.46–26.86)
Having to punish other children	117 (50.0)	101 (75.9)	16 (15.8)	16.96 (8.66–33.18)
Having to fight	232 (92.1)	100 (75.8)	22 (22.0)	11.35 (6.06–21.28)
Being forced to engage in sexual contact	79 (33.8)	60 (45.1)	19 (19.0)	3.67 (1.99–6.78)

Abbreviations: OR: odds ratio; CI: confidence interval; IDP: internally displaced persons.

^a^Data are expressed as No. (%) of respondents unless otherwise indicated. Sample sizes vary because of item-level missing data.

^b^Calculated by *χ* ^2^ test or *t* test.

^c^Odds ratios are from logistic regression models with child soldier status and sex as independent variables and traumatic exposure as the dependent variable.

**Table 2:** Total scores of Ugandan youth on measures of PTSD, psychological distress, and emotional and behavioral problems^a^.

Mental health problem and measure	Total (*n* = 234)	Former Child Soldiers (*n* = 133)	Never Abducted Children (*n* = 101)	Girls (*n* = 112)	Boys (*n* = 122)	Group Effect^b^	Gender Effect^b^
PTSD symptoms (IES-R)	51.5 (17.6) [0–88]	56 (15.2) [19–88]	45.7 (18.9) [0–84]	50.5 (17.6) [0–86]	52.5 (17.6) [0–88]	<.001	.425
Psychological distress (BSI-18)	40.9 (16.1) [0–72]	45.2 (14.6) [0–72]	35.3 (16.2) [0–72]	41.8 (16.0) [0–72]	40.2 (16.1) [0–72]	<.001	.396
Emotional and behavioral difficulties (SDQ)	16.5 (5.3) [4–29]	18.1 (5.0) [5–29]	14.4 (4.9) [4–26]	17.1 (5.2) [5–29]	15.8 (5.3) [4–28]	<.001	.018

Abbreviations: PTSD: posttraumatic stress disorder; IES-R: Impact of Events Scale Revised; BSI-18: Brief Symptom Inventory 18; SDQ: Strength and Difficulties Questionnaire. Interactions of group × gender were not statistically significant and thus are not reported.

^a^Data are expressed as mean (SD) [range].

^b^ *P* value by ANCOVA (adjusting for age as covariate).

**Table 3:** Pearson correlation coefficients of traumatic exposures and mental health problems among former child soldiers and never-abducted children.

	PTSD		Psychological distress		Emotional and Behavioral Problems	
	(IES-R)		(BSI-18)		(SDQ)	
	Former child	Never-abducted	Former child	Never-abducted	Former child	Never-abducted
	soldiers	children	soldiers	children	soldiers	children
Living in camp for IDPs	−.02	.23	.01	.18	.11	.06
Father dead	−.09	.16	−.12	.08	.01	−.03
Mother dead	−.08	.12	−.02	−.02	.01	−.12
Having to carry heavy loads	.14	.27^a^	−.05	.19	−.01	.05
Being seriously beaten	.05	.26^a^	.11	.32^a^	.03	.14
Getting injured	−.02	.33^a^	.04	.35^a^	−.03	.06
Witnessing someone being killed	.12	.28^a^	.17	.37^a^	.14	.14
Killing someone personally	.08	.16	.16	.30^a^	.20	.05
Having to drink urine	.12	.12	.29^a^	.26^a^	.30^a^	.25
Having to loot properties	−.01	.20	.03	.16	.12	.09
Having to punish other children	.06	.17	.07	.30^a^	.12	.16
Having to fight	.07	.07	−.01	.15	−.02	.15
Being forced to engage in sexual contact	.08	.20	.08	.40^a^	.06	.14
Age at abduction	−.03		−.07		−.07	
Length of abduction (months)	−.05		.04		.03	

Abbreviations: PTSD: posttraumatic stress disorder; IES-R: Impact of Events Scale Revised; BSI-18: Brief Symptom Inventory 18; SDQ: Strength and Difficulties Questionnaire.

^a^ *P* < .01, 2-tailed.

## Abbreviations

SD: Standard deviation
PTSD: Posttraumatic stress disorder
OR: Odds ratio
CI: Confidence interval
LRA: Lord's Resistance Army
IDP: Internally displaced person
IES-R: Impact of Event Scale-Revised
BSI 18: Brief Symptom Inventory 18
GSI: Global Severity Index
SDQ: Strength and Difficulties Questionnaire.
